# Enteroviral infections in the pathogenesis of type 1 diabetes: new insights for therapeutic intervention

**DOI:** 10.1016/j.coph.2018.07.006

**Published:** 2018-12

**Authors:** Sarah J Richardson, Noel G Morgan

**Affiliations:** Institute of Biomedical & Clinical Science, University of Exeter Medical School, Exeter, Devon, UK

## Abstract

The development of islet autoimmunity and type 1 diabetes has long been linked with enteroviral infection but a causal relationship has proven hard to establish. This is partly because much of the epidemiological evidence derives from studies of neutralising antibody generation in blood samples while less attention has been paid to the pancreatic beta cell as a site of infection. Nevertheless, recent studies have revealed that beta cells express specific enteroviral receptors and that they can sustain a productive enteroviral infection. Importantly, they can also mount antiviral responses which attenuate viral replication and may favour the establishment of a persistent enteroviral infection. Together, these responses combine to create the Trojan horse by which enteroviruses might precipitate islet autoimmunity.

## Highlights

• Enteroviral infection has been long-associated with type 1 diabetes in epidemiological studies.
• β-Cells express a specific enteroviral receptor isoform, CAR-SIV, mainly on secretory granules.
• β-Cells respond to enteroviruses by allowing the establishment of a persistent infection.
• Enteroviral vaccines are under development that might be effective in type 1 diabetes.

## Graphical abstract

**Figure:**
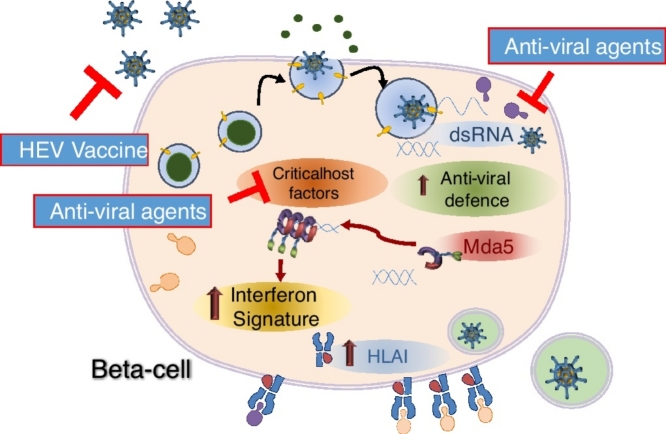

**Current Opinion in Pharmacology** 2018, **43**:11–19

This review comes from a themed issue on **Endocrine & metabolic diseases**

Edited by **Shanta J Persaud** and **James E Bowe**

For a complete overview see the Issue and the Editorial

Available online 29th July 2018

**https://doi.org/10.1016/j.coph.2018.07.006**

1471-4892/© 2018 The Authors. Published by Elsevier Ltd. This is an open access article under the CC BY license (http://creativecommons.org/licenses/by/4.0/).

Type 1 diabetes (T1D) is characterised by the selective loss of insulin producing beta cells from the islets of Langerhans in the pancreas, meaning that affected individuals must administer exogenous insulin throughout their lives. The incidence of the disease is increasing [1] and although previously considered to be predominantly a disease of the young, it is now known to develop in all decades of life [2^••^]. As a consequence, there are likely to be significant numbers of older individuals with T1D who have been mis-diagnosed with Type 2 diabetes (T2D). These two observations suggest that the number of people affected by T1D may be larger than previously thought.

Type 1 diabetes arises from a complex interaction between genetic, immune and environmental factors which, as emphasized in comprehensive recent reviews [3,4] are poorly understood. In particular, the environmental influences have proven hard to identify, although studies as far back as the 1960s have implicated viral infection, particularly by human enteroviruses (HEV; single-stranded RNA (+) viruses from the picornavirus family), as a potentially important factor both in the triggering of islet autoimmunity and the onset of clinical disease. In support of this, a 2011 meta-analysis of 26 earlier studies provided evidence that enteroviral infection occurred 3.7 times more commonly in individuals with islet autoimmunity and was 9.8 times more likely at disease onset when compared to matched controls [5]. Since that time, additional studies have emerged to support this hypothesis [6]. In particular, evidence that enterovirus infections are more frequent prior to the appearance of islet autoantibodies has been found in several large prospective cohort studies [7, 8, 9, 10]. Importantly, recent studies of unique pancreas biopsy samples from Norwegian patients with T1D (the DiViD samples [11]) have provided strong evidence for both the presence of HEV and enhanced islet anti-viral responses in newly-diagnosed patients [12,13^•^,14^•^,^15^]. In addition, ever more sensitive technologies are being developed to detect or interrogate viral infection and anti-viral responses in blood [8,16^••^,^17^, ^18^, ^19^, ^20^], islets [12,21^•^,22^•^], stool [9] and other tissues [23, 24, 25, 26, 27, 28, 29, 30, 31]. These are currently being applied in new collaborative studies involving multiple laboratories who are employing differing expertise and complementary technologies to examine blinded tissue samples available from the network of Pancreatic Organ Donors with Diabetes (nPOD). The first results are due for publication soon and are expected to provide additional support for the enteroviral hypothesis in T1D.

## Enteroviruses and beta cells: an unfortunate conjunction

Human beta cells are known to be susceptible to infection with HEVs, particularly members of the Coxsackievirus B family. Thus, isolated human islets can be productively infected with a range of different EV-B family members (CVBs and Echoviruses, many of which have been associated with T1D; Table 1) *in vitro*. Furthermore, among the various islet cells, it is the beta cells that are preferentially susceptible to infection [32, 33, 34], and this leads to a dramatic decrement in glucose-induced insulin secretion [21^•^,35^••^,^36^]. Tropism of HEVs for the islets has also been demonstrated *in vivo* in the pancreata of neonates who died following a lethal CVB infection [6,34,37] and in the pancreas of individuals with T1D [38,39]. This then raises the question: `so why the beta cells?’

**Table 1:** Examples of enterovirus serotypes associated with Type 1 diabetes or which have the ability to infect human islets *in vitro*

	Reference
General EV	[9,12,18,82, 83, 84, 85, 86, 87, 88]
CVB1-6	[7,8,32,33,35^••^,^38^,^39^,^89^, ^90^, ^91^, ^92^, ^93^, ^94^, ^95^, ^96^]
Echovirus 3, 4, 6, 9, 16, 30	[91,97, 98, 99, 100, 101, 102, 103, 104, 105, 106, 107]
Coxsackie A	[107]

**Figure 1:**
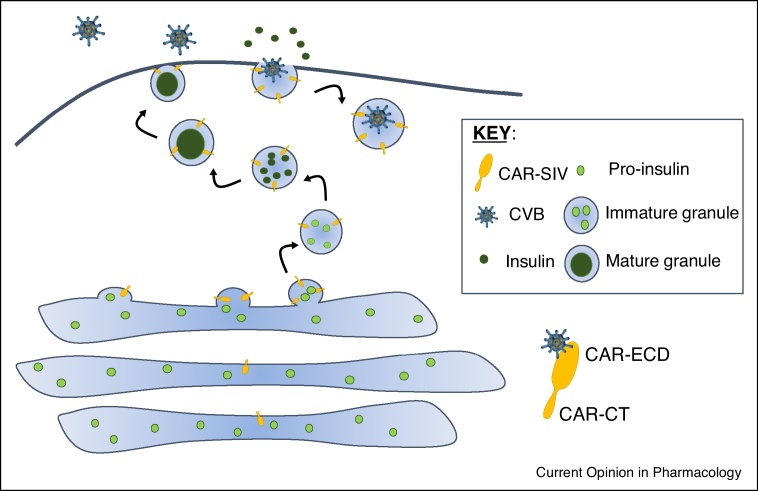
A model for CVB entry into beta cells via a specific CAR isoform, CAR-SIV. Recent data demonstrate that CAR-SIV is present at high levels on insulin secretory granules. Based on its structural organization, we predict that the C-terminus (CT) of CAR-SIV faces the cytoplasmic environment and importantly, the putative `extracellular domain’ (ECD) which is required for the binding of enteroviruses, faces the granule lumen during biogenesis and maturation. This suggests that during exocytosis of insulin, the extracellular domain of CAR-SIV will be displayed on the external face of the plasma membrane and would then be available to bind to enteroviruses. During subsequent endocytosis of the granule membrane for recycling, the virus would be transported inside the cell, where it could initiate infection.

The tropism of enteroviruses for the beta cell is likely to be driven by at least two factors; first, these cells express receptors necessary for the binding and subsequent internalisation of the virus and secondly, they contain specific host factors which the virus can hijack to facilitate successful infection, replication and, perhaps, persistence. This latter point is interesting since the traditional view states that enteroviruses are not likely to establish persistent infections and this concept will be explored further below. The various potential receptors utilised by enteroviruses and their expression in human islets are summarised in Table 2 but one that is receiving particular attention is the Coxsackie and Adenovirus Receptor (CAR). This molecule is utilised as an entry vehicle by many of the viruses that are associated with T1D in epidemiological studies and, very recently, we have shown that a specific isoform of CAR, having a unique C-terminal PDZ binding domain (CAR-SIV) is selectively and highly expressed within the beta cell [40^••^]. Studies by Ylipaasto *et al.* have also demonstrated that infection of human islets with CVB4 and CVB5 was effectively prevented in the presence of an antibody that blocks CAR [41]. Intriguingly, in our work, the subcellular localisation of CAR-SIV was unusual in that it was not present primarily at the plasma membrane of beta cells, as might be expected, but rather it was located mainly in insulin secretory granules. This unexpected localisation implies that the virus could selectively enter the beta cell by a Trojan horse mechanism in which secretory granule proteins are hijacked as they emerge onto the cell surface during exocytosis, such that virus particles are then internalized by the endocytic machinery during membrane recovery (Figure 1). In support of this, electron microscopy studies by Frisk *et al.* of human islets infected with CVBs clearly show the presence of viral replication complexes and newly synthesised virions at, or near, insulin granule membranes [35^••^].

**Table 2:** Relevant enteroviral receptors and their expression in human beta cells/islets

Potential Enterovirus receptors and *role* [78]	Enteroviruses that utilise these receptors	Transcriptomic data suggesting expression in beta cells^a^	Protein expression in islets^b^
CAR *Uncoating*	Coxsackievirus B1-6	+++	+++ [40^••^]
DAF (CD55) *Attachment*	Coxsackievirus A21, B1, B3 & B5 Echovirus 3, 6, 7, 11–13, 20, 21, 25, 29, 30	++	HPA — not detected; [41]
ICAM1 *Uncoating*	Coxsackievirus A13, A18, A21 Rhinovirus Major group (91 serotypes)	Low	HPA — not detected in healthy controls; some evidence of upregulation in inflamed T1D islets [108]
ICAM5 *Uncoating*	Enterovirus D68	Negative	HPA — not detected
SCARB2 *Uncoating*	Enterovirus 71 Coxsackievirus A16	+++	HPA — ++
PSGL1 *Attachment*	Enterovirus 71 Coxsackievirus A16	Negative	HPA — not detected
α2β1 (VLA2) *Attachment*	Echovirus 1, 8	*ITGA2* — negative *ITGB1* — +++	HPA — not detected
α5β3 *Attachment*	Coxsackievirus A9, Echovirus 1, 9	*ITGA5* — negative *ITGB3* — negative	HPA — not detected + in isolated islets [41]

CAR, Coxsackie and adenovirus receptor; DAF, complement decay accelerating factor; ICAM1, intercellular adhesion molelcule-1; SCARB2, scavenger receptor class B member 2; PSGL1, P-selectin glycoprotein ligand 1; VLA2, very late antigen 2.

a *Source*: Transcriptomics of human islets. http://sandberg.cmb.ki.se/pancreas/.

b *Source*: Human Protein Atlas (HPA) or references. https://www.proteinatlas.org/.

In recent years, a series of critical host factors required for successful HEV infections have been identified. These include PLA2G16 [42^••^] which is essential for virion-mediated genome delivery into the cytoplasm; phosphatidylinositol-4-kinase IIIβ (PI4KIIIβ) and its product phosphatidylinositol-4-phosphate (PI4P), which are critical for the generation of specialised organelles required for efficient viral replication [43,44]); polypyrimidine tract-binding protein 1 (PTBP1), which is utilized by the virus to promote cap-independent translation of viral RNA [36] and heat shock protein 90 (HSP90), which is required for the correct processing of the capsid precursor P1 [45]. Importantly, many of these proteins are expressed in human beta cells and play a key role in pathways unique to beta cells (reviewed in [46]). For example, PTBP1, has an important role in glucose-stimulated cap-independent translation of insulin granule proteins [36]; PI4KIIIβ acts as a metabolic sensor in beta cells and regulates the priming of secretory granules [47] and HSP90 is a chaperone that regulates surface expression of ATP-sensitive potassium (K_ATP_) channels [48]. Together these results suggest that human beta cells express specific virus entry receptors as well as key host factors that aid the virus at various points in its lifecycle. This may therefore help to explain their exquisite sensitivity to infection.

**Figure 2:**
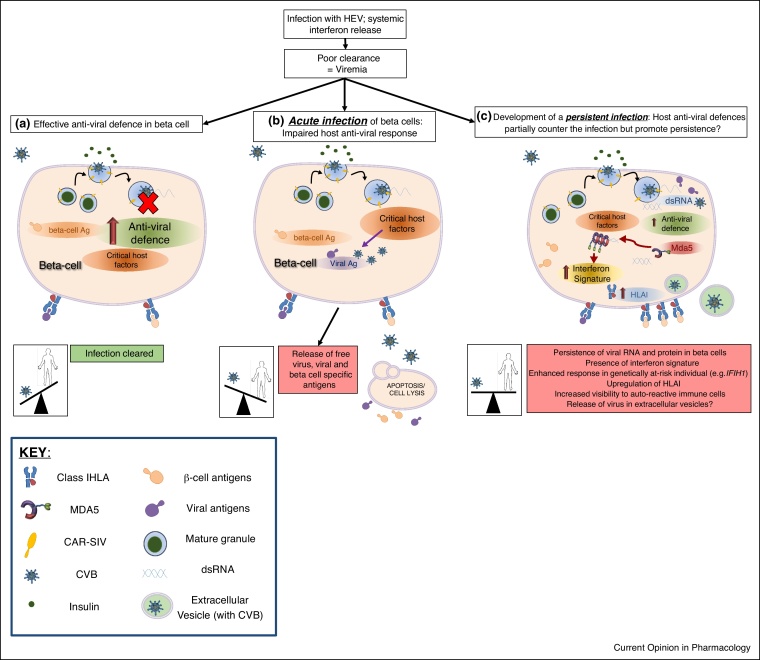
A model of different beta cell responses to HEV infection. Following an infection with a HEV, systemic release of interferons primes the pancreas to respond to the likelihood of a local viral infection. **(a)** In most individuals this will lead to the induction of an anti-viral defence program which prevents the development of a sustained and productive infection of the beta cells. The virus is cleared and the host wins the battle. **(b)** In some individuals (possibly neonates?) who have an impaired anti-viral defence, enterovirus enters the cells and utilises critical host factors to establish a productive, lytic, infection. This can result in the release of free virus and/or viral and beta cell specific antigens. In individuals who are genetically predisposed to T1D, this damage may trigger the activation of islet autoreactive immune cells. **(c)** If the host anti-viral defence program only partially inhibits viral replication, then a persistent infection might develop. Persistent infections are associated with 5′UTR deletions of the viral genome and the formation of dsRNA. dsRNA can activate host pathogen recognition receptors (PRRs) such as Mda5 (encoded by *IFIH1*) and stimulate an enhanced interferon signature in cells. This will, in turn, lead to the upregulation of HLAI and enhanced presentation of beta cell and viral antigens at the cell surface. In `at-risk’ individuals this might then result in destruction by auto-reactive immune cells. Virus could be disseminated to other cells via extracellular vesicles, although this remains to be determined for human beta cells.

In parallel with these considerations, it is also important to note that beta cells are terminally differentiated and studies of their neogenesis and proliferation suggest that these processes are vanishingly rare in humans after the age of 10y [49,50]. This means that the human host must develop strategies to effectively manage, or preferably clear, any beta cell viral infection, whilst doing everything possible to minimise the destruction of these largely irreplaceable cells. In this context, it is well known that beta cells are extremely sensitive to interferons (IFNs), the principal anti-viral cytokines produced in response to an infection [51,52]. As viremia (viruses in the bloodstream) must occur before infection of the beta cells it is likely that any cells targeted during the initial acute phase of infection will elaborate IFNs. Thus, the pancreas will be exposed to IFNs prior to any encounter with the virus and this may serve to prime the beta cells to resist infection. Indeed, pre-treatment of islets with Type I and III IFNs promotes an anti-viral state and significantly reduces viral replication following infection *in vitro* [51,52]. However, IFNs do not necessarily block viral entry, which could yield a scenario in which virus has entered the cell, yet the host cell has succeeded in upregulating a range of anti-viral proteins that will counter any attempt by that virus to establish a productive, lytic, infection [53]. Conceivably, a battle then ensues between the host (beta) cell and the virus which culminates in a mutual compromise where viral persistence is established and the host cells remain viable (Figure 2). In support of this hypothesis, risk-associated single nucleotide polymorphisms (SNPs) for T1D are found in key anti-viral response genes such as *IFIH1* and *TYK2*. Individuals carrying these SNPs exhibit altered IFN responses [54, 55, 56, 57, 58, 59, 60,61^••^] and the risk variants have been associated with an increased frequency of HEV infection [62]. Furthermore, of the 51 identified candidates genes associated with T1D, 42 are expressed in human beta cells and when Ingenuity Pathway Analysis was performed on these genes, the three highest scoring canonical pathways were — *Interferon signalling*, *Role of JAK1, JAK2 and TYK in interferon signalling* and *Role of pattern recognition receptors in recognition of virus and bacteria* [54]. These pathways are all activated in response to viral infections and this provides a possible link between genetic predisposition to T1D and host anti-viral responses.

## Evidence for persistence of HEV infection

Traditionally, HEVs are thought to induce an acute infection in which large numbers of new viral particles are rapidly synthesized and release from infected cells to spread to other nearby host cells. One of the most effective host mechanisms to control HEVs infection is the generation of neutralising antibodies which help to clear the virus from the circulation and affected tissues. However, there is mounting evidence that HEVs can evade these primary defense mechanisms to establish a lower level, persistent, infection under certain circumstances. Indeed, this type of infection has now been associated with several diseases such as Chronic Fatigue Syndrome (CFS) [63]; chronic myocarditis and dilated cardiomyopathy [64] and Amyotrophic Lateral Sclerosis (ALS; reviewed in [65]) as well as Type 1 diabetes. In order to understand this previously unrecognized aspect of EV biology, a number of mechanisms have been proposed to explain how the virus might persist. These include the activation of processes to restrict viral RNA replication, including by deletion of nucleotides from within the 5′UTR of the viral RNA genome [64,66, 67, 68] and equalization of the proportions of positive and negative strands to form double stranded RNA (dsRNA) molecules [69,70]. A key additional requirement is the need for the virus to minimise host cell lysis, which would not only promote inflammation and the activation of antiviral immune cells, but also lead to the release of free virus particles that are susceptible to neutralization by anti-EV antibodies. One mechanism by which this may be avoided is suggested by recent evidence that HEVs, including CVB3, can be shed from cells within extracellular vesicles [71,72]. In principle, this could shield the virions from neutralizing antibodies and provide a means by which they can evade immune surveillance.

## Tackling HEV infection in individuals with, or who are at-risk of developing, T1D

Two main strategies are being explored to tackle HEV infection in T1D; vaccination and treatment with anti-viral agents. Both have the potential to slow disease progression, yet each also has significant obstacles that must be overcome before it could be utilized in clinical practice. Islet autoimmunity in at-risk children peaks at two different ages and the specificity of the first autoantibody also differs at each age [73]. The first peak of autoimmunity occurs during the second year of life and is associated with the development of insulin autoantibodies (IAA), while the second is seen between 3 and 5y and is associated preferentially with the emergence of GADA autoantibodies [73]. Given that these initial signs of islet autoimmunity occur early in life, children would probably need to be vaccinated within the first few months of life in order to offer effective protection against HEV infection. This will require the development of safe and effective vaccines that can target multiple HEVs associated with the disease and there is precedent for this approach given the proven success of neonatal vaccination against poliomyelitis (another enterovirus). Moreover, encouraging progress has already been made on this front, with a new formalin-inactivated CVB1 vaccine successfully developed and tested in animal models [74,75^••^,^76^]. Multivalent CVB1-6 vaccines are also now being generated (Hytönen and Flodström-Tullberg, personal communication). Epidemiological data support the idea that a vaccination approach might be effective as a means to reduce the incidence of T1D since Finnish children infected early in life with CVB3 or CVB6 appear to be immuno-protected against a subsequent infection with different HEVs which might, otherwise, precipitate T1D [7]. On the basis of such evidence, one company, `Provention’ has recently announced exciting plans to launch a first phase clinical trial to assess the safety and efficacy of a CVB vaccine in humans, with the intention of developing an effective approach to reduce the incidence of T1D (www.proventionbio.com).

Alternative approaches also being explored include the development of virus like particles (VLPs) as antigens. These resemble the viral capsid of HEVs but do not contain infectious genome [77]. Vaccines and/or VLP rely predominantly on the host developing neutralizing antibodies against virus. These will therefore be most effective when given to individuals prior to any exposure to diabetogenic viruses and will hopefully ensure that the immune response is sufficiently robust to prevent the spread of infectious virus to the pancreas. What, though, might be done to tackle infection in people who already have evidence of islet autoimmunity and/or clinical diabetes and who may be harbouring a persistent infection?

This could be a fertile realm for anti-viral agents; although at the present time very little is known about whether these are effective against persistent enteroviral infections. Anti-viral agents are available (many of which have been re-purposed from use in other conditions), or are in development, that are effective against HEVs associated with T1D (comprehensively reviewed recently in [78]), but the majority of these have been tested only under acute infection settings. These agents can be subdivided into two broad categories; those that target viral proteins and others which affect host proteins required for efficient viral replication, translation and release. Examples of the former include pleconaril which targets the viral capsid (reviewed in [79]); fluoxetine, commonly known as Prozac, which inhibits the viral protease 2C; and Gemcitabine, which binds to the viral RNA-dependent RNA polymerase, 3D^pol^ [80]. The second group includes Enviroxime, which targets the PI4K pathway [79]. Encouragingly, recent evidence has suggested that fluoxetine is effective against persistent enteroviral infection in cell models [81], but more research is required to test the activity of other drugs in this setting. Extensive efforts are underway to identify new anti-viral agents and these are aided by an increasing knowledge of the structure and function of viral proteins, as well as the identification of essential host factors. A further avenue of exploration is the use of combinations of different anti-viral agents that have additive or synergistic responses, which together can increase anti-viral potency, minimise the emergence of resistance and reduce drug toxicity/side effects. This could be achieved by, for example, combining one drug that targets a viral protein, with another that targets an essential host factor. Alternatively, since it is well established that some anti-viral drugs have a low barrier to resistance (meaning that a single mutation within the virus can rapidly lead to drug resistance) whereas, for others, this is much higher, a combination approach employing each type of reagent might also yield clinical benefit.

In summary, evidence for a viral aetiology in T1D is a long-established concept that has remained unproven. Nevertheless, supportive evidence continues to emerge at increasing pace and effective strategies which would minimize the risks deriving from HEV infection in susceptible individuals are being developed with increasing momentum. Arguably, it is only when the outcomes of these studies are known that it will be possible to confirm once-and-for-all whether T1D has an enteroviral component.

## Funding

We are pleased to acknowledge financial support via a JDRF Career Development Award (5-CDA-2014-221-A-N) to SJR, an MRC Project Grant (MR/P010695/1) to SJR & NGM and project grants from Diabetes UK (15/0005156 & 16/0005480) to NGM & SJR.

## Conflict of interest statement

Nothing declared.

## Author contributions

S.J.R and N.G.M. wrote the manuscript and are the guarantors of this work.

## References and recommended reading

Papers of particular interest, published within the period of review, have been highlighted as:

• of special interest
•• of outstanding interest
